# A case-control study of medium-term exposure to ambient nitrogen dioxide pollution and hospitalization for stroke

**DOI:** 10.1186/1471-2458-13-368

**Published:** 2013-04-19

**Authors:** Julie YM Johnson, Brian H Rowe, Ryan W Allen, Paul A Peters, Paul J Villeneuve

**Affiliations:** 1 Population Studies Division, Health Canada, Ottawa, Canada; 2 Department of Emergency Medicine, Faculty of Medicine and Dentistry and School of Public Health, University of Alberta, Edmonton, Canada; 3 Faculty of Health Sciences, Simon Fraser University, Burnaby, Canada; 4 Health Analysis Division, Statistics Canada, Ottawa, Canada; 5 Dalla Lana School of Public Health, University of Toronto, Toronto, Canada

**Keywords:** Stroke, Air pollution, Odds ratio, Case-control study, Land use regression, NO_2_

## Abstract

**Background:**

There are several plausible mechanisms whereby either short or long term exposure to pollution can increase the risk of stroke. Over the last decade, several studies have reported associations between short-term (day-to-day) increases in ambient air pollution and stroke. The findings from a smaller number of studies that have looked at long-term exposure to air pollution and stroke have been mixed. Most of these epidemiological studies have assigned exposure to air pollution based on place of residence, but these assignments are typically based on relatively coarse spatial resolutions. To date, few studies have evaluated medium-term exposures (i.e, exposures over the past season or year). To address this research gap, we evaluated associations between highly spatially resolved estimates of ambient nitrogen dioxide (NO_2_), a marker of traffic pollution, and emergency department visits for stroke in Edmonton, Canada.

**Methods:**

This was a case-control study with cases defined as those who presented to an Edmonton area hospital emergency department between 2007 and 2009 with an acute ischemic stroke, hemorrhagic stroke, or transient ischemic attack. Controls were patients who presented to the same emergency departments for lacerations, sprains, or strains. A land-use regression model provided estimates of NO_2_ that were assigned to the place of residence. Logistic regression methods were used to estimate odds ratios for stroke in relation to an increase in the interquartile range of NO_2_ (5 ppb), adjusted for age, sex, meteorological variables, and neighborhood effects.

**Results:**

The study included 4,696 stroke (cases) and 37,723 injury patients (controls). For all strokes combined, there was no association with NO_2._ Namely, the odds ratio associated with an interquartile increase in NO_2_ was 1.01 (95% confidence interval {CI}: 0.94-1.08). No associations were evident for any of the stroke subtypes examined.

**Conclusion:**

When combined with our earlier work in Edmonton, our findings suggest that day-to-day fluctuations in air pollution increase the risk of ischemic stroke during the summer season, while medium term exposures are unrelated to stroke risk. The findings for medium term exposure should be interpreted cautiously due to limited individual-level risk factor data.

## Background

The evidence of ambient air pollution effects on cardiovascular morbidity and mortality is growing [1-12]. There are indications that exposure to fine particulate air pollution can exert influences on the long-term development of atherosclerotic plaques [13,14], short-term decreases in heart rate variability [15] and increases in vasoconstriction [16]. Levels of fibrinogen, a pro-coagulant in plasma, increase over the short-term and remain elevated over the long-term following exposure to NO_2_, a gaseous pollutant strongly associated with vehicular traffic [17]. While many population-based studies have examined short-term effects of ambient air pollution on vascular outcomes, including stroke, there are fewer studies of longer term effects [18]. Among cohort studies of prolonged exposure to ambient air pollution on risk of stroke, there is inconsistency in results [3,11,12,19].

We have previously investigated the relationship between ambient air pollution levels and hospitalization for stroke in Edmonton using different exposure measures and study designs. Short term (i.e., day to day) elevations in NO_2_ (ppb) in Edmonton were associated with an increased risk of ischemic stroke between April to September and those with certain co-morbidities (i.e., diabetes, history of heart disease, history of previous stroke) were more susceptible to the effects of these short term elevations [7,20]. While we have investigated the association between a longer term measure of air pollution and stroke using an ecologic study format [21], the spatial resolution of the air pollution measures was relatively coarse (neighborhoods of 1,600 to 54,000 households). In this study, we evaluate the association between ambient air pollution and emergency department visits using a land-use regression (LUR) model to assign highly resolved estimates of NO_2_. The LUR models allow us to assign estimates of air pollution concentrations at a resolution of less than 50 m to the residential address of the study subjects. Unlike our case-crossover studies that rely on fixed site monitoring data, the land use regression estimates better reflect exposure to ambient concentrations of NO_2_ that occur over a longer period. The rationale for conducting this study was to investigate whether a medium term measure of exposure to NO_2_ (i.e., the past year) that captured within-urban area variations in exposure was associated with emergency department visits for stroke.

## Methods

### Patient data

This was a case-control study with the case series being those individuals who presented to Edmonton area hospitals for stroke, while the control series were those who presented to the same hospitals for a series of conditions deemed to be unrelated to air pollution. The data for cases and controls were obtained from emergency department (ED) administrative data collected from 11 hospitals in and around Edmonton, Alberta. Eligible visits occurred between January 1, 2007 and December 31, 2009. Case patients comprised those with most responsible diagnoses coded by International Classification of Diseases, 10^th^ version (ICD-10) I60x through I64x, and G45x. We classified strokes into three mutually exclusive groupings: hemorrhagic (I60-62), acute ischemic (I63-64), and transient ischemic attack (TIA, G54).

Ideally, the selection of a control series would have been done from Edmonton residents who had never experienced a stroke event. Practically, it was not possible to create this control series by randomly selecting Edmonton residents and asking them to participate in the study. The costs of doing so were prohibitive, and more importantly, based on previous Canadian experiences, participation rates for this type of recruitment would be modest (~50-60%) therefore making it nearly impossible to determine whether participating controls would be a representative study population. Instead, we opted to create a control series of patients using hospitalization data. Namely, controls consisted of those patients who visited one of the 11 EDs in the Edmonton area during the study period for lacerations to head, arm, hand, leg, or foot (ICD-10:S01, S41, S51, S61, S71, S81, S91), sprains/strains to leg, foot (S83, S93), and injury to muscles or tendons of ankle or foot (S96). As outlined by Rothman and Greenland, the major problem of using hospital controls occurs when they are not selected independently of exposure in the source population [22]. However, we are confident that this is not the case given that there is no reason to believe that long-term exposure to ambient air pollution is associated with a higher incidence of lacerations particularly given that all such cases would have been treated in hospitals and not in physician offices or walk-in clinics. Hospital control series have commonly been used to investigate effects of air pollution for cardiovascular disease [23-26].

**Figure 1:**
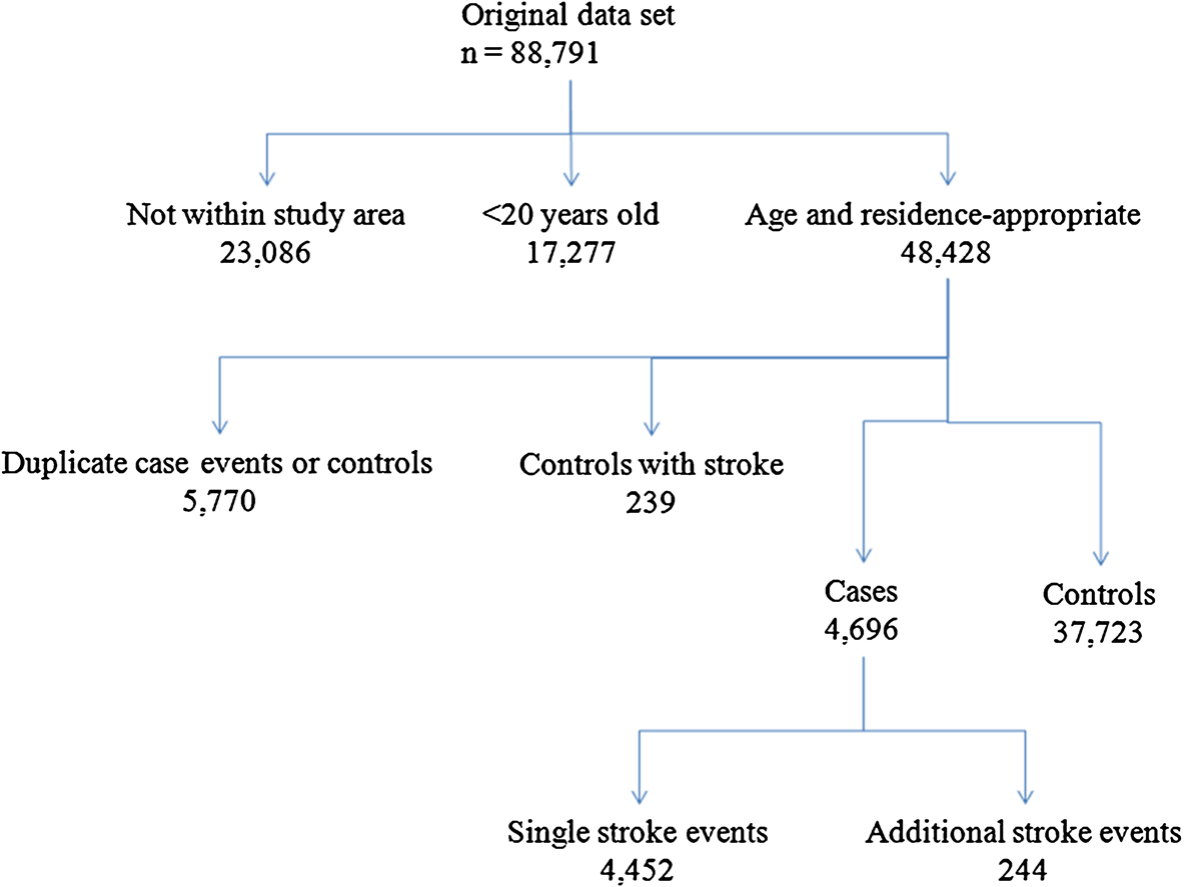
Flow chart of study population.

Edmonton ED sites receive stroke patients transferred from central or northern Alberta and other patients needing emergency care living in neighboring communities that lack these services. Twenty-six percent of individuals in the original dataset (n = 88,791) did not have residential postal codes within Edmonton. Since exposures could not be readily assigned for these people, and because these patients were not part of our target population they were removed from analyses (Figure 1). Twenty years of age was used as the lower age limit for cases and controls as it has been applied elsewhere to define young adult stroke [27-29]; in applying this exclusion criterion, 19.5% of patients in the original dataset were dropped. Duplicate admissions were excluded for a) any case who was admitted with the same stroke type as in a previous admission or b) for controls with >1 control admission during the study period (5,770, 11.9% of 48,433). Individuals who were eligible to be classified as both case and control were counted in the study dataset as a case only and were not included as a control (5.1% of 4,696 case patients), to ensure that all controls were non-cases during the study period. During the study period, 244 patients (4.8% of all case events) visited an Edmonton ED at different times with diagnoses of different stroke type (Figure 1). For these individuals, each stroke type diagnosis was recorded as a separate event to be included in each of the corresponding stroke type stratum. Ethics approval for this study was granted by the Health Research Ethics Boards of the University of Alberta and Health Canada.

### Exposure data

Ambient concentrations of NO_2_ (ppb) were estimated from a LUR model for the city of Edmonton [30] (Figure 1). The model was developed based on geographic data and measurements with Ogawa passive samplers at 50 locations during two 14-day sampling campaigns (January 27 to February 10, 2008 and April 27 to May 11, 2008). The final model included variables that classified proximity to surface water, industrial zones, city centre, all roads, major roads, and highways. The spatial resolution was less than 50 meters and the model R^2^ was 0.81. We used the LUR values at the centroid of each 6-character postal code in Edmonton, and merged these values to the residential postal codes for each patient at the time of the ED visit. In urban areas, the 6-character postal code typically indicates a specific block (one side of a street between 2 intersecting streets), a single building or sometimes a large volume mail receiver.

### Socioeconomic data

Contextual measures of socioeconomic status (SES) corresponding to the neighborhoods lived in by the patients were obtained from the 2006 Canadian Census. The census tract (CT) of residence was determined using Statistics Canada’s postal code conversion software (PCCF+ Version 5J) [31]. Five SES variables were constructed at a CT level for each case and control. These included the proportions of residents who a) lacked a high school certificate, b) had a university degree, c) were in the lowest neighborhood income quintile, and d) were in the highest neighborhood income quintile. Multiple ecologic measures for education and income reduces the potential for misspecification of group-level data [32].

Neighborhood income quintiles were based on the published average income per single person equivalents in the CT. Neighborhood income quintiles were calculated using person weights from the Statistics Canada low-income cut-offs (LICOs) to derive a single-person equivalent multiplier by household size. Population LICO quintiles were calculated for the Edmonton CMA rather than nationally, as housing costs vary substantially across Canada and area-based quintiles better reflect income adequacy relative to need [33,34]. Data on smoker type and body mass index (BMI) for Edmonton residents were obtained from the Canadian Community Health Survey (CCHS), years 2001, 2003, 2005, and 2007 [35] to investigate the possibility that smoking and BMI, two risk factors for stroke, are independently associated with NO_2_.

### Statistical analyses

Pearson’s correlation tests were conducted to evaluate the association between age, SES variables and interquartile range (IQR) increases in NO_2_. Given the potential for non-linear associations between age and exposure or outcome, patients were categorized into five age- groupings: 20-34, 35-49, 50-64, 65-79, ≥80 years. Logistic regression models were fitted to estimate the risk of all strokes, transient ischemic attacks, acute ischemic attacks, and hemorrhagic strokes in relation to an IQR increase in NO_2_. All models were adjusted for sex and age-group. Fully adjusted models included sex, age, and all contextual SES variables. To evaluate the potential for residual confounding due to smoking and body mass index (BMI), CCHS data were merged with the LUR NO_2_ data by postal code, and analysis of variance and least significant difference method were used to test for differences in mean NO_2_ between groups of Edmonton residents categorized by self-reported smoker status and by quartiles of BMI.

Upon selection of the control series, we noted that their age distribution was different from that of the case series. We conducted additional sensitivity analyses to evaluate what effect, if any, this age imbalance had on our findings. We did this in two ways. First, we restricted analysis to include only study subjects who were 65 years of age and older. Second, we randomly selected a control series that was individually matched to the case by age (within 10 years), and performed conditional logistic regression to evaluate associations between air pollution and stroke. All models were generated in PASW (SPSS-IBM) 18.0 (Armonk, NY). Comparisons of risk estimates between models using Cochran’s Q were run in R [36].

## Results

**Figure 2:**
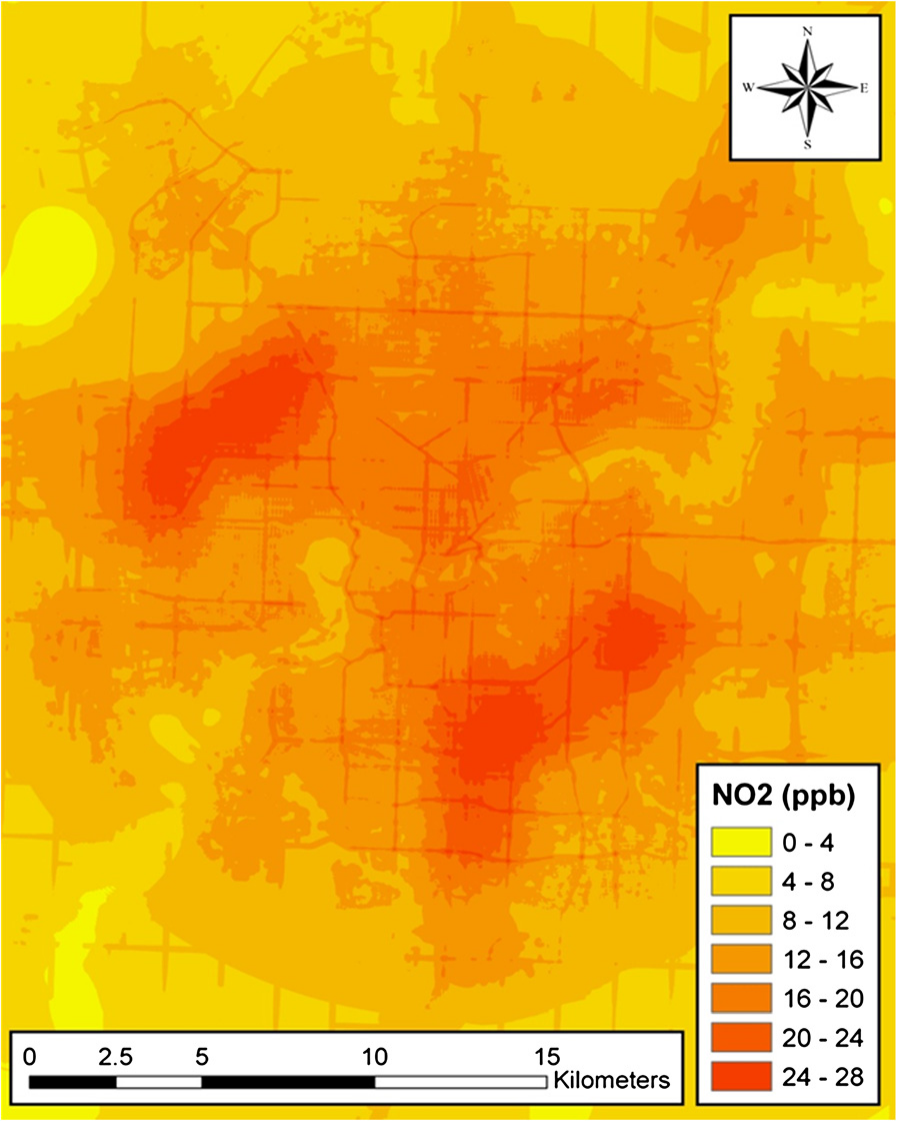
**Land use regression-derived NO**_**2 **_**surface for Edmonton, Alberta, 2007 **[30]**.**

**Figure 3:**
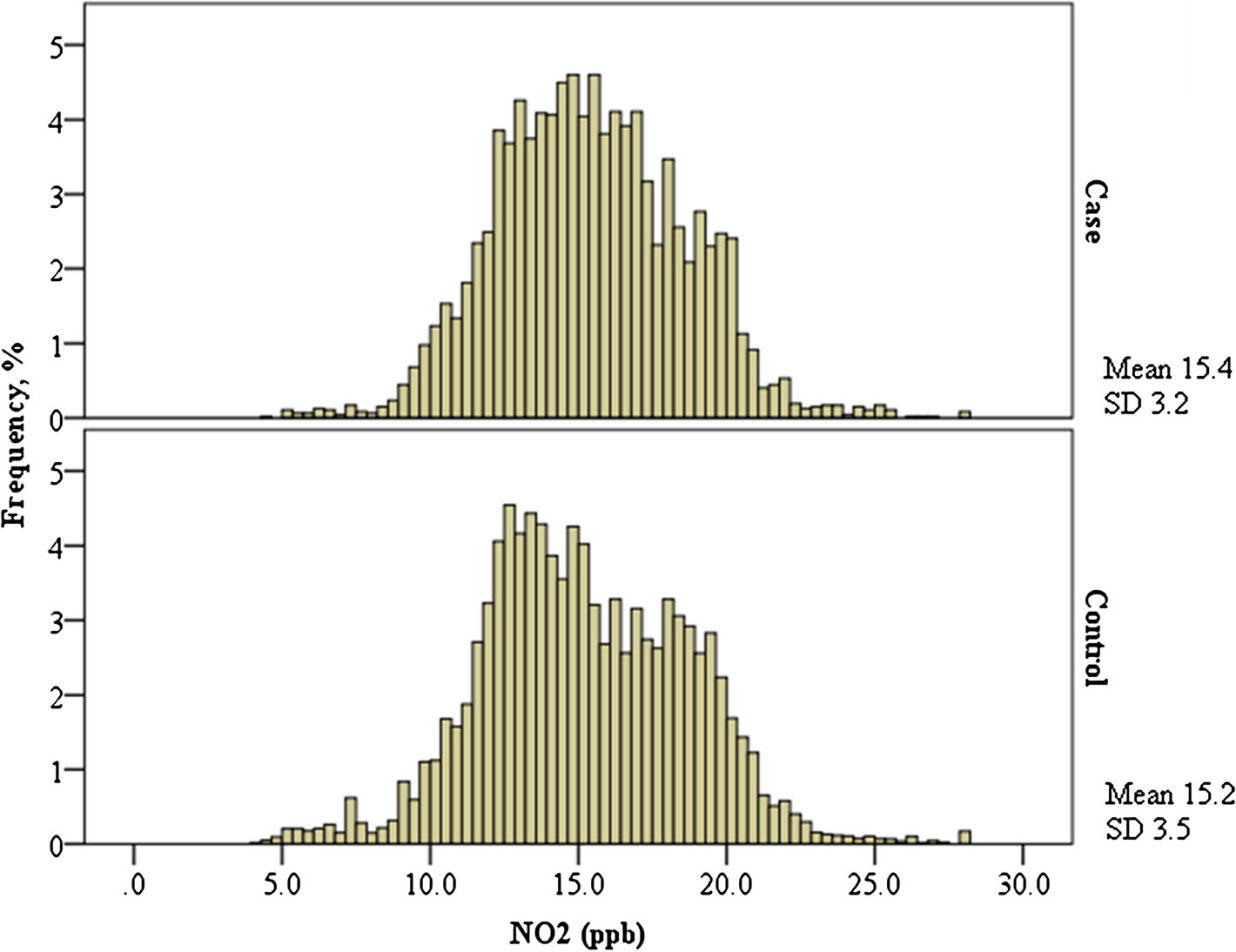
**Distributions of NO**_**2 **_**exposure levels comparing cases (top panel) to controls (bottom panel), Edmonton, Alberta.** 2007-2009, percent of panel totals.

The case series consisted of a total of 4,696 stroke events presented to Edmonton area hospitals with stroke. Among these individuals there were 2,224 acute ischemic strokes, 1,736 TIAs, and 736 hemorrhagic strokes. There were a total of 37,724 controls identified during this same period. Figure 2 displays the variation in ambient concentrations of NO_2_ across the Edmonton urban area. The mean concentration of NO_2_ obtained from the land-use regression among cases was 15.4 ppb (standard deviation=3.2) (Figure 3). The corresponding values in the control series were similar (mean=15.2 ppb, standard deviation=3.5 ppb); the mean concentration among cases was 1% higher than it was among controls (*t* = 3.96, *p* <0.001). On average, cases were older than controls and there was a higher proportion of males than females among controls (Table 1).

**Table 1:** Sample population from emergency department patients, Edmonton, Alberta, 2007-2009

	Control^*^	**Acute ischemic stroke**^**†**^	**Hemorrhagic stroke**^**†**^	**Transient ischemic attack**^**†**^	**All strokes**^**†**^
N	37,724	2,224	736	1,736	4,696
Males, number (%)	24,382 (64.6)	1,128 (50.7)	404 (54.9)	792 (45.6)	2,324 (49.5)
Females, number (%)	13,341 (35.4)	1,096 (49.3)	332 (45.1)	944 (54.4)	2,372 (50.5)
Age, mean years (SD)	39.8 (16.9)	70.9 (15.0)	66.3 (17.4)	69.7 (14.6)	69.7 (15.3)
25^th^ Percentile NO_2_, ppb	12.8	13.3	13.3	12.9	13.1
Median NO_2_, ppb	14.9	15.4	15.4	15.1	15.3
75^th^ Percentile NO_2_, ppb	17.8	17.8	18.0	17.2	17.5

^*^ Control group: Laceration, strain, sprain, patients, ICD-10 codes S01, S41, S51, S61, S71, S81, S91, S83, S93, S96.

^†^ Case group: Stroke patients, ICD-10 codes: transient ischemic attacks (G45x), acute ischemic attacks (I63x, I64), and hemorrhagic strokes (I60x, I61x, and I62x). Individual patients may have more than one type of stroke during study period.

There were no statistically significant associations between ambient concentrations of NO_2_ and any stroke outcome (Table 2). The fully-adjusted OR for all strokes (combined) in relation to an IQR increase in ambient NO_2_ was 1.01 (95% CI: 0.94-1.08), while the corresponding estimates for acute ischemic and hemorrhagic strokes were 1.02 (95% CI: 0.94-1.13), and 1.07 (95% CI: 0.92-1.24), respectively. The influence of SES increased the risk estimates slightly, however, not to the point of achieving statistical significance. There was no evidence of heterogeneity in the odds ratios when stratified analysis was conducted by sex. Specifically, the fully-adjusted OR associated with an IQR increase in NO_2_ for all strokes was 0.99 (95% CI: 0.89-1.09) for women, while it was 1.03 (95% CI: 0.93-1.14) for men.

**Table 2:** Odds ratios of stroke in relation to a control series of injury patients in relation to an increase of 5 ppb^* ^**in NO**_**2**_**, Edmonton, Alberta, 2007-2009**

	**Adjusted for sex, age**		**Adjusted for sex, age, SES**^**†**^	
Stroke category	OR	95% CI	OR	95% CI
All stroke	0.99	0.94-1.04	1.01	0.94-1.08
Acute ischemic	1.02	0.95-1.10	1.03	0.94-1.13
Transient ischemic attack	0.89	0.82-0.96	0.95	0.86-1.05
Hemorrhagic	1.07	0.96-1.20	1.07	0.92-1.24

^*^ Corresponding to the interquartile range for NO_2_.

^†^ Includes high school completion, university degree, lowest income quintile membership, highest income quintile membership.

There was significant variability in NO_2_ levels among groups of Edmonton residents based on smoking type from the CCHS data (analysis of variance, *F* = 5.7, *p* = 0.001), but levels of NO_2_ exposure among the daily smoker group (mean = 15.7 ppb) was only 3% higher than the levels of never smokers (mean = 15.2 ppb) (Table 3). No association was observed between BMI and NO_2_ exposure levels (data not shown, available upon request).

**Table 3:** **NO**_**2 **_**exposure (ppb) and smoker type, Edmonton, Alberta (Canadian Community Health Survey data, 2001, 2003, 2005, 2007)**

			***p*-value for least significant difference**		
	**n (%)**	**Mean NO**_**2**_**(SD)**	**Daily**	**Occasional**	**Former**
Daily	1,249 (20.9)	15.70 (3.24)			
Occasional	283 (4.7)	15.52 (3.63)	0.40		
Former	2,127 (35.6)	15.42 (3.29)	0.02	0.63	
Never	2,308 (38.6)	15.23 (3.19)	<0.001	0.16	0.05

In comparison to the full data set risk estimate (1.01; 95% CI: 0.94, 1.08), the fully-adjusted OR corresponding to an IQR increase in NO_2_ for the data set restricted to ages >65 years was slightly lower, 0.98 (0.90, 1.07). Risk estimates for the 3 stroke subtypes shifted similarly downwards (data not shown). When the controls were matched to cases, the ORs shifted upwards for all strokes (1.03; 95% CI: 0.94, 1.12). There were no significant differences in comparisons of risk estimates.

## Discussion

The primary objective of our study was to investigate associations between ambient concentrations of NO_2_ and the risk of stroke using a spatially refined measure of air pollution that was available from a land use regression model. This study did not evaluate effects of fine particulate matter for which both short term and long term exposures have been associated with the risk of stroke [2,37]. Concentrations of NO_2_, to a far greater extent than fine particulate matter, vary considerably within intra-urban areas, and are regarded as a surrogate measure of traffic related air pollution [38]. Our previous analyses of the associations between day to day changes in air pollution and stroke revealed associations that were dominated by NO_2_ during the summer months [20].

In this case-control study, we found no association between an IQR increase in LUR-derived measures of NO_2_ and stroke. LUR models are based on land use and traffic patterns within urban centers and can provide high resolution estimates of within-city ambient NO_2_ concentrations. In comparison to exposures estimated from interpolation models of air pollution where monitor locations are sparse, data from an LUR generally have less spatial error [39,40]. In this respect, our present work uses a much more refined measure of exposure than we applied in our previous ecologic analysis of stroke risk and long-term exposure to ambient NO_2_ in Edmonton. In that ecological study we used an interpolation model of air pollution based on data from two fixed-sited monitoring stations [21], and no association with stroke was found for either NO_2_ or fine particulate matter (PM_2.5_) after adjusting for other contextual confounding variables. So, even with the improvements of individual-level data and higher spatial resolution, we were still unable to find a longer-term effect of pollution (medium- or long-term) on stroke events in Edmonton. In contrast, we found positive associations between day to day increases in NO_2_ and ischemic stroke risk in Edmonton during the summer months [7,20]. The difference may be due to a greater pathogenetic influence of air pollution exposure on processes involved in triggering stroke over the short-term compared to the slow progression of atherosclerosis or venous thrombosis over a longer time frame [18]. Combined, the findings from across our studies suggests that only day to day elevations in ambient air pollution, but not medium term exposures, increase the risk of stroke. While findings from the case-control study should be interpreted with some caution given the lack of data on individual level risk factors, we found very little difference in NO_2_ levels among current and never smokers in the CCHS.

Elsewhere, cohort studies published to date have generated an inconclusive body of evidence on the effects of long-term exposures on stroke. While an association with an IQR increase in NO_2_ was reported in a cohort analysis in Denmark (fully adjusted hazard ratio = 1.05; 95% CI: 0.99-1.11) [3], no association was found with a 10 μg/m^3^ increase in particulate matter <2.5 μm in aerodynamic diameter (PM_2.5_) among a 10-year cohort study in Canada [12]. However, data from a cohort of women in the US found that stroke events were strongly associated with a 10 μg/m^3^ increase in PM_2.5_ (hazard ratio: 1.28, 95% CI: 1.02-1.61) [11] and a positive association was also observed with a 10 μg/m^3^ increase in PM_10_ in the California Teachers cohort (1.06, 95% CI: 1.00,1.13) [19]. Although we note that our exposure data were not historical and we were not modeling long-term effects on stroke risk, we did not find a similar effect of NO_2_ on stroke among women in our case-control population.

In contrast to cohort studies, case-control studies lack the ability to examine time-at-risk effects; however, they may offer improvements in cost and statistical efficiencies. Our study findings align closely those from a recent case-control study of acute strokes in Scania, Sweden where no association was found between stroke and 10 μg/m^3^ increases in NO_x_ (OR = 0.93; 95% CI: 0.82-1.95) [1]. That study and ours differed in regards to the patient population. While their case series pooled ICD-10 codes I61x, I63x, and I64x [41], our overall dataset of stroke patients also included those with discharge diagnoses I60x, I62x, and G54x. Our total stroke dataset represent a more heterogeneous set of clinical outcomes; however, these data permitted us to further consider the possibility that air pollution exerts different effects on clinically different stroke types.

In our analyses, we assumed medium-term residency at the postal code given in the hospital database, based on the observation that 18.1% of Edmonton residents reported moving residence within the previous 12 months, in the 2006 Census [42]. Given the association between age and stroke and the higher tendency to move among younger Canadians compared to older residents [43], even medium-term exposure misclassification could have been more common among controls than cases.

It appears that even with high spatial resolution exposure data, our results do not differ substantially from previous studies; there is no evidence of a medium-term effect of NO_2_ on risk for any subtype of stroke, or overall stroke. In the only study of short-term effects of NO_x_ on TIA, Henrotin *et al* found no association between 10 μg/m^3^ increases in NO_x_ and TIA (OR = 0.86; 95% CI: 0.74, 1.02) [6]. Similar to our present findings, we found no association between 5-year average concentration of NO_2_ and TIA in our previous ecologic analysis of stroke in Edmonton [21]. Hemorrhagic stroke risk is unaffected by long-term ambient NO_2_ exposure [3,21,44]; on this point our results are also consistent with those from other studies.

Our study is, to our knowledge, the first case-control study of air pollution effects on stroke to use hospital controls. While a population-based control group would be more representative of the source population, such controls are not readily available in Canada. Regardless, our use of hospital controls was methodologically sound. Because there are no plausible biological mechanisms to suggest that long term exposure to ambient pollution increases the risk of experiencing lacerations, odds ratios were not affected by this type of Berksonian bias [45]. Traffic density is associated with traffic injury, but patients suffering from those events are coded as ICD-10 V01 – X59 [46]; thus, our control group, patients presenting with lacerations, strains, sprains would not have been involved in events associated with exposure.

In case-control studies, selection bias may also arise when a confounder influences risk estimates and the distribution of the confounder between case and control groups arises due to control group selection [47]. Age was strongly associated with case status, weakly and variably associated with exposure, and was controlled for in our models. We conducted 2 sensitivity analyses which suggest that these models sufficiently controlled potential confounding due to the difference between age distributions of case and control groups. Hospital-based control sampling ensured that the controls would have had access to the study EDs had they had stroke and allowed us to eliminate patients who were stroke and laceration patients during the study period. We feel any residual confounding may have less impact on our results than would any self-selection bias that is inherent in population-based control sampling with low response rates [48]. Strokes are more common among women than among men and admissions for accidental wounds, strains, and sprains may be more common among men [49,50]. We controlled for the confounding due to sex in our analysis, thereby reducing potential selection bias inherent in our control group.

Increased risk for ischemic stroke among smokers has been documented extensively and reviewed recently [51]; however, with group-level data we did not find that NO_2_ was strongly different across smoking groups. Oudin *et al.* observed stronger effects of NO_x_ on acute strokes among non-smokers than among smokers [52]. It is possible, then, that smoking could have been an effect modifier of our results. Data on other known risk factors for stroke such as diabetes, hypertension, and previous stroke [53] could have also allowed us to define sub-populations with increased susceptibility to the effects of ambient air pollution, but these data were not available among hospital administration databases. Data on anti-coagulant use would help us to determine those who have been controlling their risk of stroke, if they already have known risk factors for cardiovascular disease or stroke. Also, without historical medical data, we cannot rule out the additional possibility that individuals with existing risk factors for stroke, including chronic diseases, chose to move closer to high-traffic areas of Edmonton to shorten travel times to specialized health care. With regionalization of stroke and cardiac care units into higher level tertiary facilities [54], generally, located close to universities, there may be unintended harmful effects on those populations with potentially greater vulnerability to air pollution effects. Our study, however, included patients from 11 hospitals of various levels of specialization (primary, secondary, and tertiary care) throughout the city, so, the potential for intensive health care needs among a higher stroke-risk group to cause selection bias is minimal.

When socioeconomic variables that captured small area effects of education and household income were entered into the models they produced only a small increase in the odds ratios for most outcomes. However, there is an inherent limitation due to the reliance on CT-level data for SES indicators. In metropolitan centers, CT-level data may poorly represent individual-level deprivation [55]. However, while the association between health and SES is stronger at the individual level, the direction of the association is the same at the ecological level. The probability of misclassification could be argued to be greater among those in areas of higher traffic density, as the population density within the CT would be greater than in areas at the city boundary.

## Conclusion

In this case-control study, the high-resolution data generated by the LUR model provided us with highly accurate and precise estimates of NO_2_ exposure in the Edmonton area; nevertheless, we found no increased risk of stroke, acute ischemic stroke, TIA, or hemorrhagic stroke associated with NO_2_. This adds to the increasing body of evidence against medium- and long-term effects of ambient air pollution on stroke risk, in which, it appears that, in general, short-term stroke-triggering effects from exposure to ambient air pollution are relevant than effects from longer term exposure.

## Abbreviations

LUR: Land use regression; CT: Census tract; LICO: Low-income cut-off; ED: Emergency department; TIA: Transient ischemic attack; NO2: Nitrogen dioxide; OR: Odds ratio; CI: Confidence interval; IQR: Inter-quartile range; ICD: International classification of diseases; SES: Socioeconomic status; BMI: Body mass index; SD: Standard deviation

## Competing interests

The authors declare that they have no competing interests.

## Authors’ contributions

JYMJ participated in study design, conducted analyses, and drafted and revised the manuscript. PJV originated study concept, participated in study design, advised analyses, and revised the manuscript. BHR participated in the study design, supervised data collection, and revised the manuscript. RWA provided LUR model data and revised the manuscript. PAP provided the SES data and revised the manuscript. All authors read and approved the final manuscript.

## Pre-publication history

The pre-publication history for this paper can be accessed here:

http://www.biomedcentral.com/1471-2458/13/368/prepub
